# RURAL-URBAN DISPARITIES IN UNMET LONG-TERM CARE NEED AND COMMUNITY CARE SERVICES EXPECTATION AMONG ELDERLY IN CHINA

**DOI:** 10.1093/geroni/igz038.569

**Published:** 2019-11-08

**Authors:** Yanbing Zeng, lixia wang, Ya Fang

**Affiliations:** 1 Xiamen University, Xiamen, China; 2 School of Public Health, Xiamen University, Xiamen, China

## Abstract

Objective This study aimed to examine the urban-rural differences of unmet needs and their expected LTC services among community-dwelling old people. Methods The data comes from the Chinese Longitudinal Health Longevity Survey (CLHLS) in 2014. A total of 1587 community residents aged 65+ with disability of activities of daily life (ADL) were included in this study. Binary logistic regression was used to estimate correlates of unmet need in LTC. And chi-square test was used to examine the differences of expected community-based LTC services between urban and rural area. Results Over half (55.07%) of the participants reported their need were unmet. For both rural and urban residents, poorer economic status and reluctant caregivers (ORs>1, P＜0.01) seriously affected the unmet need. Besides, of urban older adults, people who were male and lonely(ORs>1, P＜0.05) reported more unmet need. While of rural old ones, people who were with severe ADL disability and poorer self-rated health(ORs>1, P＜0.01) reported more unmet need. And people with available medication and home visit services(ORs<1, P＜0.01) reported more met need. However, the supplies for community LTC care services were far below the demands. Conclusion The risk of having unmet need associated with ADL disabilities in LTC is largely determined by their economic status and caregivers’ willingness to provide care for both rural and urban old people. There is a need for an overall improvement in the planning, provision and financing of long-term care services for elderly individuals in China.

